# Risk prediction of echinococcosis in China under climate change: a One Health approach

**DOI:** 10.1186/s13071-026-07518-7

**Published:** 2026-06-18

**Authors:** Tengyue Diao, Nan Xu, Ning Ma, Haiyan Chen, Na Wang, Ting La, Kunyi Wu, Bo Cao

**Affiliations:** 1 Department of Infectious Diseases, The Second Affiliated Hospital of Xi’an Jiaotong University, Xi’an, 710004 China; 2 Core Research Laboratory, The Second Affiliated Hospital of Xi’an Jiaotong University, Xi’an, 710004 China; 3 Department of Clinical Laboratory, The Second Affiliated Hospital of Xi’an Jiaotong University, Xi’an, 710004 China; 4 National-Local Joint Engineering Research Center of Biodiagnosis and Biotherapy, The Second Affiliated Hospital of Xi’an Jiaotong University, Xi’an, 710004 China; 5 Key Laboratory of Surgical Critical Care and Life Support (Xi’an Jiaotong University), Ministry of Education, Xi’an, 710004 China

**Keywords:** Echinococcosis, Risk prediction, One health, Climate change, Maxent model, Parasitosis

## Abstract

**Background:**

Echinococcosis, a neglected zoonosis caused by *Echinococcus* parasites, imposes a dual burden on public health and socioeconomic development across China, with disproportionate impacts on impoverished pastoral communities. A critical barrier to targeted and effective control lies in the lack of high-resolution national risk maps and limited understanding of how climate change modulates transmission dynamics.

**Methods:**

Here, we address these gaps by integrating multi-source epidemiological and environmental data to model and map high-resolution echinococcosis transmission risk across China. We further project its spatiotemporal evolution under four climate change scenarios (SSP126, SSP245, SSP370, SSP585) from 2040 to 2100, employing a One Health framework to assess echinococcosis transmission risk. Our model exhibits robust predictive performance, identifying elevation, annual precipitation, precipitation seasonality, isothermality, and average monthly precipitation in January as key driving factors.

**Results:**

Results reveal concentrated high-risk regions in western and northern China, including Sichuan, Qinghai, Tibet, Xinjiang, and Gansu provinces, which are characterized by pastoral economies, socioeconomic underdevelopment, and constrained healthcare access. Future projections show a concerning expansion of high and very high transmission risk regions across all scenarios, with the most significant increase under the high-emission SSP585 pathway by the late twenty-first century.

**Conclusions:**

These findings clarify the current echinococcosis risk landscape and its environmental determinants while providing forward-looking, spatially explicit evidence. This work establishes a science-based foundation for optimizing resource allocation, designing adaptive prevention strategies, and enhancing health equity within a One Health framework, particularly for climate-vulnerable and resource-limited settings.

**Graphical Abstract:**

**Figure:**
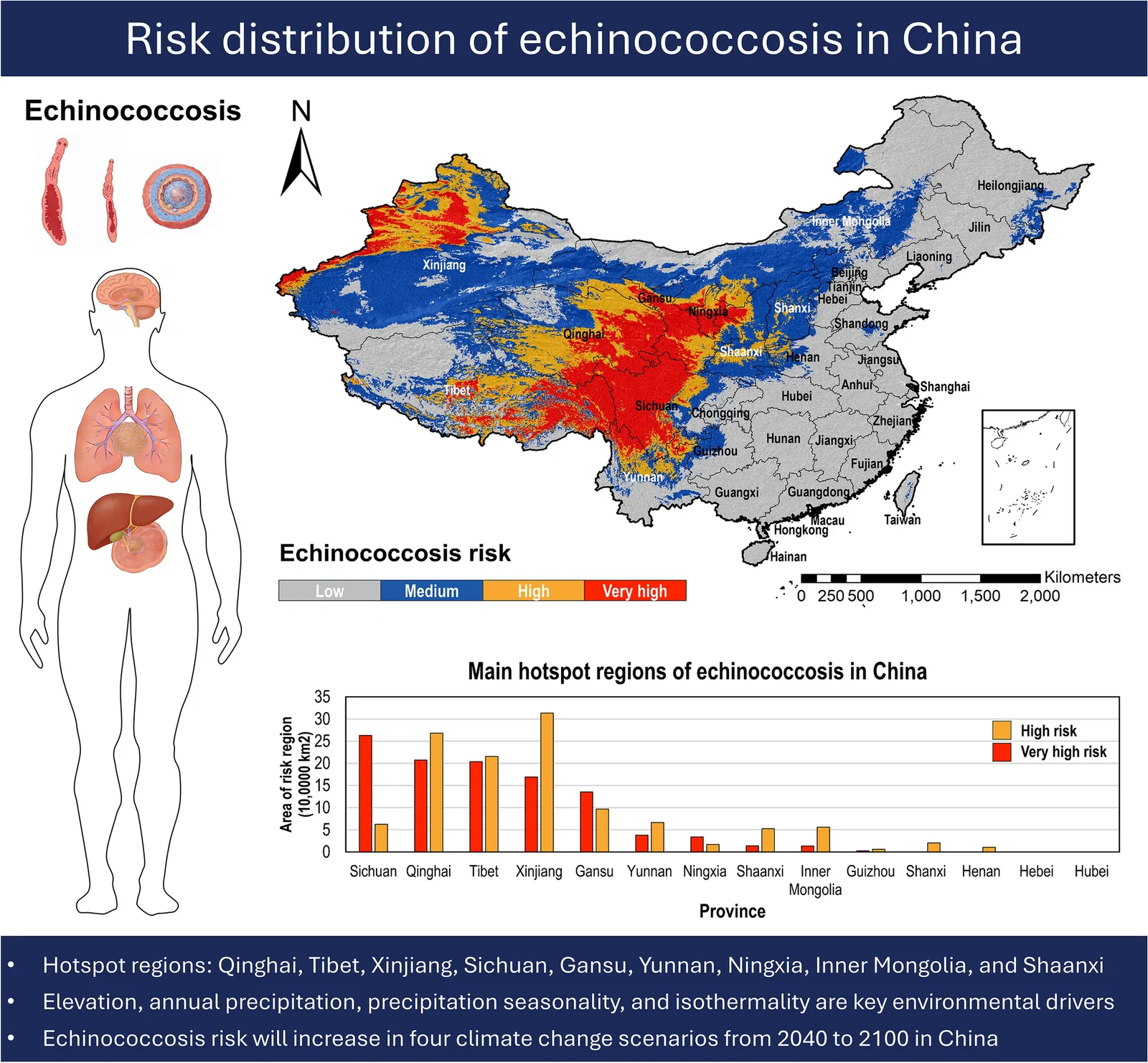

**Supplementary Information:**

The online version contains supplementary material available at 10.1186/s13071-026-07518-7.

## Background

Echinococcosis, a globally significant neglected zoonotic parasitic disease caused by the larval stages of *Echinococcus* tapeworms (specifically *E. granulosus* and *E. multilocularis*), poses a persistent threat to public health and undermines socioeconomic sustainability worldwide [1, 2]. In China, the disease is hyperendemic in western and northern pastoral regions, where crude incidence rates surge to 50–100 per 100,000 population in focal areas–rates that far exceed global averages [3, 4]. Beyond its catastrophic clinical toll-with advanced alveolar echinococcosis associated with a 5-year mortality rate of up to 94%-echinococcosis inflicts a crippling economic burden, encompassing exorbitant medical expenditures and substantial livestock husbandry losses that total over 1.3 billion RMB annually [5, 6]. This dual burden of morbidity/mortality and economic hardship disproportionately afflicts vulnerable pastoral communities, exacerbating health inequities and impeding regional development, making it a critical barrier to achieving global health and development goals.

**Fig. 1:**
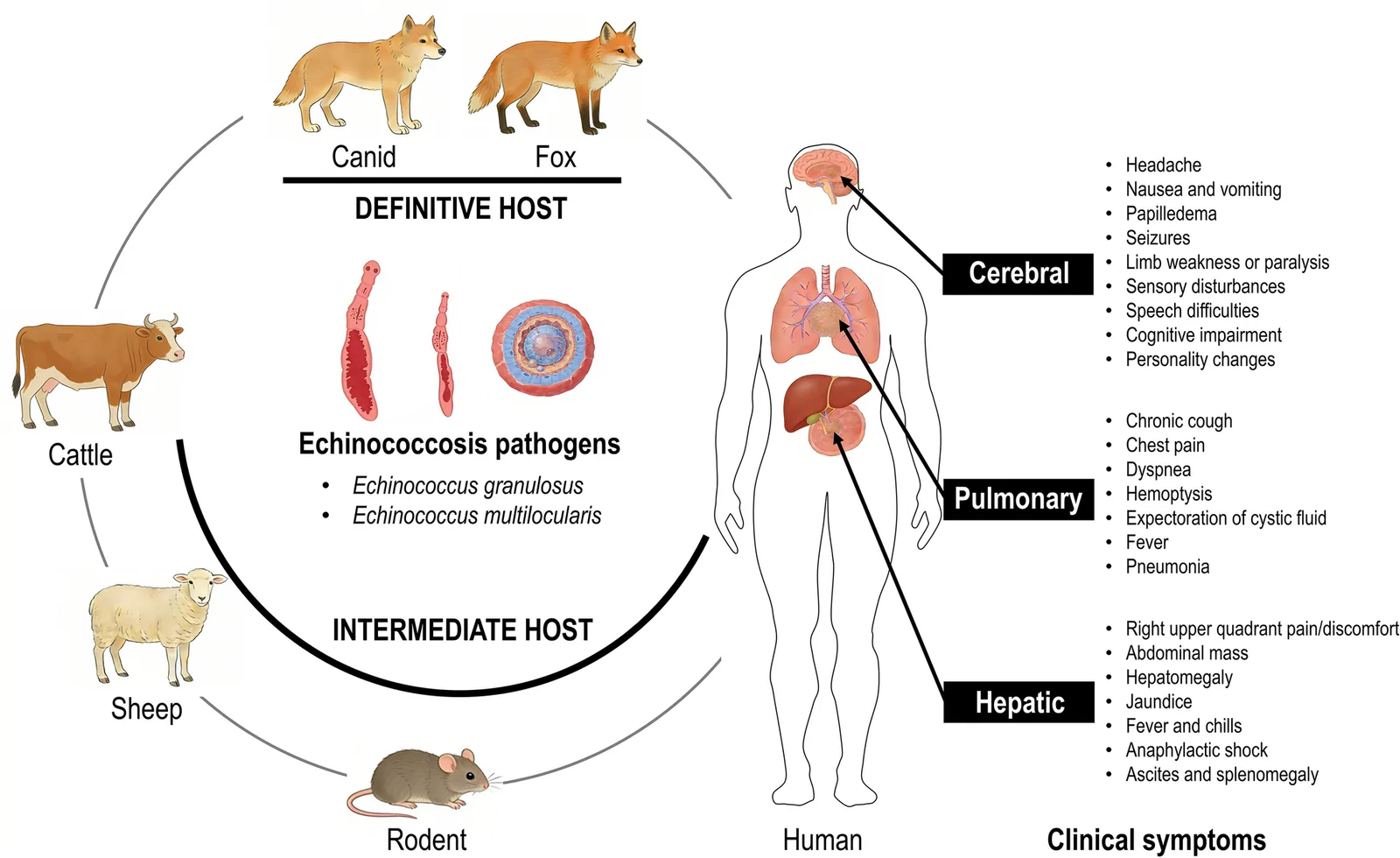
Schematic diagram of *Echinococcus* transmission chain (pathogen–host–human) and key clinical symptoms of echinococcosis. The lifecycle of *Echinococcus* begins with the definitive host (canid, fox) excreting infectious eggs into the environment. These eggs are subsequently ingested by an intermediate host, typically cattle, sheep, or rodents, where they hatch and develop into larval forms. Humans may accidentally become an intermediate host by ingesting eggs through contact with a contaminated environment. Consequently, infection can lead to key clinical manifestations, including cerebral, hepatic, and pulmonary symptoms

The transmission of echinococcosis is governed by intricate multi-trophic ecological networks involving pathogens, intermediate hosts (e.g., sheep, cattle, rodents), definitive hosts (e.g., dogs, foxes), and human populations–networks that are tightly coupled to biogeographical processes and anthropogenic activities (Fig. 1) [7–9]. Humans act as accidental intermediate hosts, acquiring infection through ingestion of *Echinococcus* eggs excreted by definitive hosts; this risk is acutely heightened for pastoralists, herdsmen, and veterinarians due to their frequent contact with infected animals or contaminated environments [10–12]. The interdependence of ecological, animal, and human health in echinococcosis transmission renders it a paradigmatic disease that demands a One Health framework to guide integrated prevention and control efforts [13, 14]. Yet, translating this One Health paradigm into actionable strategies remains hindered by incomplete understanding of how key environmental and climatic drivers modulate the structure and function of these transmission networks.

Despite sustained investments in echinococcosis control in China, two critical, inter-related knowledge gaps persist, hampering the transition from broad-based interventions to targeted, evidence-driven strategies. First, existing echinococcosis risk maps are predominantly low-resolution, failing to resolve fine-scale spatial heterogeneities in transmission risk heterogeneities that are critical for prioritizing resource allocation to high-burden foci and optimizing intervention efficiency. Second, the implications of global climate change for echinococcosis transmission dynamics remain poorly elucidated. Climate change reshapes the biogeographical ranges, survival, and reproductive success of *Echinococcus* hosts by altering temperature, precipitation, and other key environmental parameters, which in turn can restructure transmission networks and shift the epidemiological landscape of the disease [15–18]. As climate change intensifies, projecting future shifts in echinococcosis risk has become an urgent imperative for formulating proactive, climate-resilient long-term control strategies. Without such projections, efforts to mitigate the disease’s burden are likely to be reactive and ineffective in the face of evolving environmental conditions.

To bridge these critical knowledge gaps and address the pressing need for climate-informed, One Health-driven echinococcosis control, we designed this study to achieve three overarching objectives: (1) develop a high-resolution spatial framework for echinococcosis infection risk across China by integrating epidemiological incidence data and environmental covariates; (2) project spatiotemporal dynamics of echinococcosis risk under four shared socioeconomic pathways (SSP) (SSP126, SSP245, SSP370, SSP585) across four time periods (2021–2040, 2041–2060, 2061–2080, 2081–2100); and (3) provide a rigorous scientific basis for targeted echinococcosis prevention, optimized resource allocation, and climate-resilient adaptation strategies in China. By integrating multi-source data and predictive modeling within a One Health paradigm, this work advances our understanding of echinococcosis epidemiology under global change and delivers actionable technical support for China’s echinococcosis elimination initiatives—with broader implications for addressing neglected zoonoses in other climate-vulnerable, resource-limited regions worldwide.

## Methods

### Echinococcosis incidence

**Fig. 2:**
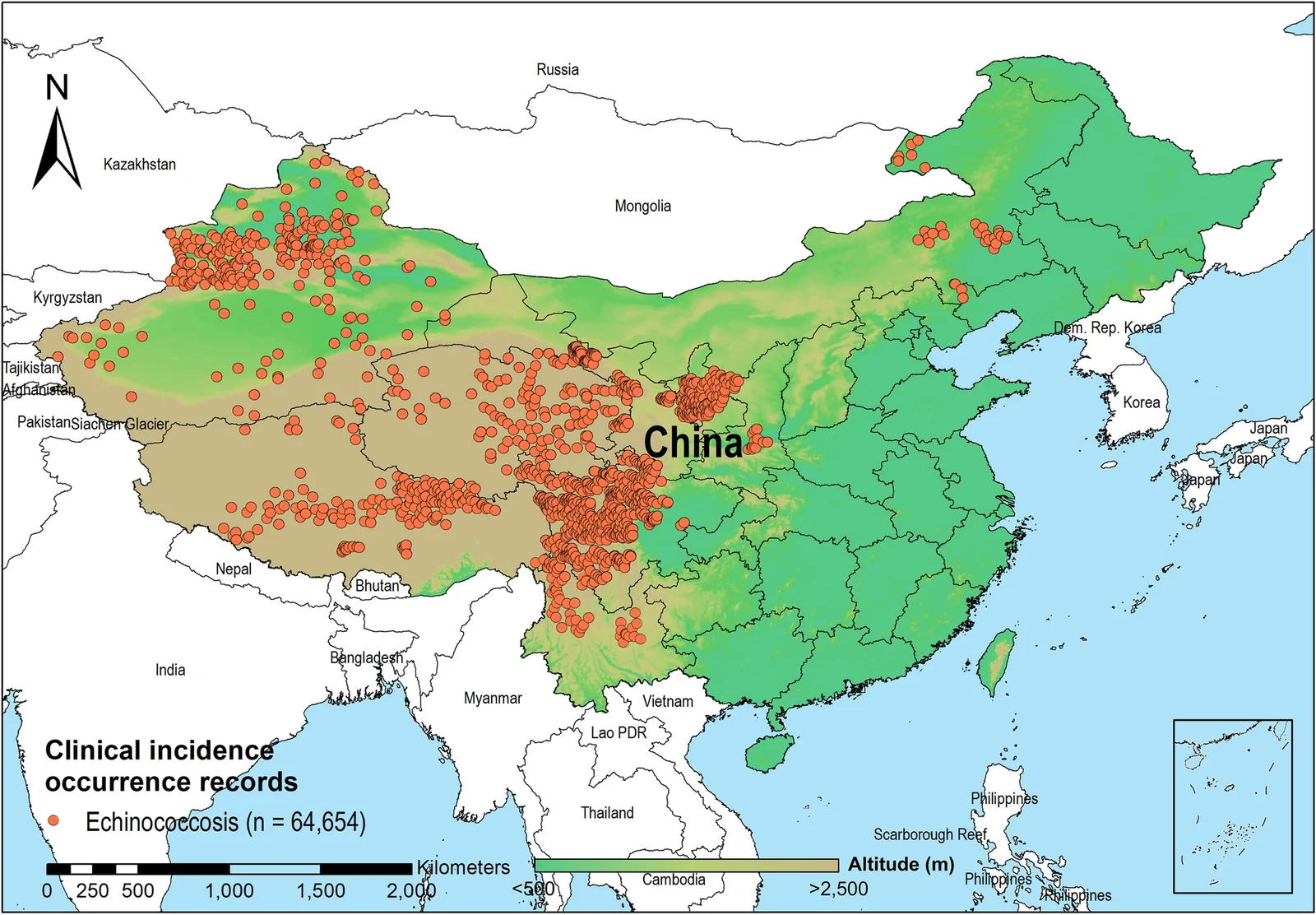
Geographical distribution map of echinococcosis incidence points in China. Echinococcosis incidence data were collected from multiple sources including structured clinical surveys, systematic searches of peer-reviewed publications, and official surveillance records and annual reports archived by the Chinese Center for Disease Control and Prevention to construct a comprehensive, nationwide dataset during the period from 2004 to 2024. A total of 64,654 validated occurrence points was used for subsequent risk modeling

Echinococcosis incidence data were collated from three major categories of sources to construct a comprehensive, nationwide dataset during the period from 2004 to 2024. These sources included: (1) structured field epidemiological and clinical surveys conducted in endemic pastoral and agro-pastoral regions across China; (2) systematic searches of peer-reviewed publications indexed in major scientific databases (PubMed, Web of Science, CNKI, Wangfang, VIP; a detailed workflow of literature review and screening process is given in supplementary Fig. S1); and (3) official surveillance records and annual reports archived by the Chinese Center for Disease Control and Prevention (China CDC, https://www.chinacdc.cn) and provincial/local health commissions. The compiled dataset covers all 34 provincial-level administrative divisions of China and includes georeferenced incidence points (longitude and latitude), annual or period-specific incidence rates, and associated demographic characteristics. In this study, we mainly adopted georeferenced human echinococcosis incidence records as presence data, which serve as the most reliable, systematically collected and long-term available dataset at the national scale. Although independent field survey datasets of definitive hosts (e.g., dogs, foxes) and intermediate hosts (e.g., sheep, cattle, rodents) were not directly incorporated as separate occurrence variables in our model, we incorporated a suite of topographic and bioclimatic environmental covariates that closely govern the habitat suitability, spatial distribution and population dynamics of definitive hosts, intermediate hosts, as well as the survival and development of *Echinococcus* parasites. These environmental proxies could indirectly reflect the ecological suitability for host survival and parasite egg persistence, and implicitly encapsulate the complete transmission linkages among definitive hosts, intermediate hosts and human populations under the One Health framework. After systematic integration, deduplication and quality screening, a total of 64,654 validated georeferenced human occurrence points were ultimately preserved for subsequent ecological niche modeling. To mitigate spatial sampling bias and autocorrelation, a spatial filtering procedure was implemented using the SDM Toolbox in ArcGIS 10.2. Incidence points were spatially rarefied by applying a minimum distance threshold of 2 km and a maximum distance threshold of 25 km, ensuring a more spatially independent distribution of records. To validate the appropriateness of spatial thinning thresholds, we performed Moran’s I spatial autocorrelation analysis across a gradient of maximum distance thresholds (10, 25, and 30 km), with a fixed minimum distance of 2 km. Results indicated that the dataset thinned with a 2 km minimum distance and 25 km maximum distance showed no statistically significant spatial autocorrelation (*P* > 0.05), confirming that the selected thresholds effectively eliminated residual spatial autocorrelation and ensured spatial independence of model input points (Table S1). Following this filtering process, the resulting set of 64,654 validated occurrence points was used for subsequent ecological niche modeling. For model training and validation, we ran 100 bootstrap replicates; in each replicate, 75% of the validated occurrence points were randomly allocated for model training, with the remaining 25% reserved as an independent testing dataset to assess model discriminatory power (Fig. 2).

### Environmental variables

Environmental variables were carefully selected and processed to ensure robust model performance and meaningful ecological interpretation. For current climate conditions, we obtained 104 high-resolution (30 arc-seconds, approximately 1 km at the equator) raster layers during the 1970–2000 baseline period from the WorldClim database (version 2.1). These layers comprised 19 bioclimatic variables, monthly temperature, precipitation, wind speed and solar radiation data, and elevation. To prevent model instability and enhance interpretability caused by multicollinearity, we performed a pairwise Pearson correlation analysis. Variables with a correlation coefficient |*r*| ≥ 0.8 were considered highly collinear. This threshold is widely accepted and commonly adopted in ecological niche modeling and zoonotic disease risk prediction [19, 20]. Compared with a stricter threshold of |*r*| ≥ 0.7 that may unnecessarily exclude biologically meaningful environmental predictors and a relaxed threshold of |*r*| ≥ 0.9 that cannot sufficiently suppress multicollinearity, the cutoff of |*r*| ≥ 0.8 achieves an optimal balance between eliminating strong collinearity and retaining ecologically critical variables suitable for the alpine pastoral ecosystem of echinococcosis transmission. From each correlated pair, we retained the variable deemed more directly relevant to the known ecology of *Echinococcus* transmission. Finally, the selection process determined a total of 11 key environmental variables for model prediction (a full list of these variables is given in supplementary Table S2).

For future climate projections, corresponding data layers for these variables were sourced from the BCC–CSM2–MR global climate model, part of the Coupled Model Intercomparison Project Phase 6 (CMIP6). This model has demonstrated good performance in simulating East Asian climate dynamics [21, 22]. Previous model evaluation and intercomparison studies have verified that BCC–CSM2–MR exhibits superior capability in reproducing the climatic features of western inland China and the Qinghai–Tibet Plateau, making it particularly suitable for projecting climate conditions across the echinococcosis endemic regions in this study. We extracted data for four future 20-year periods (2021–2040, 2041–2060, 2061–2080, 2081–2100) under four shared socioeconomic pathways (SSP126, SSP245, SSP370, SSP585). To ensure consistency and comparability with the current baseline data, all future climate projections were prepared with a 30-arcsecond resolution.

### Maxent model for risk prediction

To model the risk probability for echinococcosis transmission, we employed the Maximum Entropy (Maxent) model (version 3.3.3 k), a presence-background machine learning method widely validated for species distribution modeling and disease risk mapping [20, 23, 24]. Maxent is particularly suited for this study as it performs robustly with presence-only data and complex environmental interactions [25–27]. Model calibration involved 100 bootstrap replicates to quantify prediction uncertainty and enhance robustness. For each replicate, occurrence records were randomly partitioned, with 75% allocated for model training and the remaining 25% reserved for independent testing. All models were configured to generate a logistic output, representing a continuous probability of echinococcosis transmission risk (ranging from 0 to 1).

### Modeling evaluation

Model performance and statistical robustness were rigorously assessed using a multi-faceted validation framework. The discriminatory capacity of the Maxent model was primarily evaluated by calculating the area under the receiver operating characteristic curve (AUC) based on the independent test dataset. Model predictive accuracy was interpreted using established thresholds, where an AUC ≥ 0.8 denotes excellent performance. To confirm that model predictions were non-random and significantly better than chance, we performed a null-model validation. This involved constructing 100 null models by training Maxent with randomly generated pseudo-absence points, equal in number to the actual species occurrence records but distributed uniformly across the study extent. The significance of our actual model was determined by comparing its AUC value against the distribution of AUCs from the null models using a one-tailed *t* test, with a threshold of *P* < 0.05. Finally, to elucidate the relative influence of each environmental predictor and enhance the ecological interpretability of the model, we conducted a Jackknife test. This procedure quantified the contribution rate (reflecting the variable’s usefulness during model training) and the permutation importance (measuring the dependency of model predictions on each variable) for all retained environmental factors. To further quantify model predictive performance and consistency, the continuous boyce index (CBI) and true skill statistic (TSS) were additionally calculated based on the 25% independent test subset across all 100 bootstrap replicates. CBI was adopted to assess the model’s ability to distinguish suitable from unsuitable habitats, while TSS was used to evaluate overall predictive accuracy independent of prevalence.

### Classification and calculation of infection risk

To translate the continuous modeling output into a spatially explicit, interpretable measure of infection risk, we constructed an infection risk index (*R*). The value of this index is equivalent to the probability of the model output ranging from 0 to 1, following our previous protocols [20, 24, 26, 27]. The resulting continuous *R* values, representing the potential infection risk of echinococcosis, were then classified into four distinct tiers to facilitate visualization and policy prioritization: low risk (0 ≤ *R* < 0.1), moderate risk (0.1 ≤ *R* < 0.3), high risk (0.3 ≤ *R* < 0.5), and very high risk (*R* ≥ 0.5). This standardized, tiered classification system enables a direct and intuitive comparison among hotspot regions with different infection risks across all geographic scales.

### Statistical and spatial analysis

Spatial data processing, map drawing, and risk calculation were conducted using ArcGIS (version 10.2). Statistical analyses, including Pearson correlation analysis and significance testing, were conducted using IBM SPSS Statistics (version 23.0). Data visualization was conducted using GraphPad Prism (version 9.0).

### Study framework

**Fig. 3:**
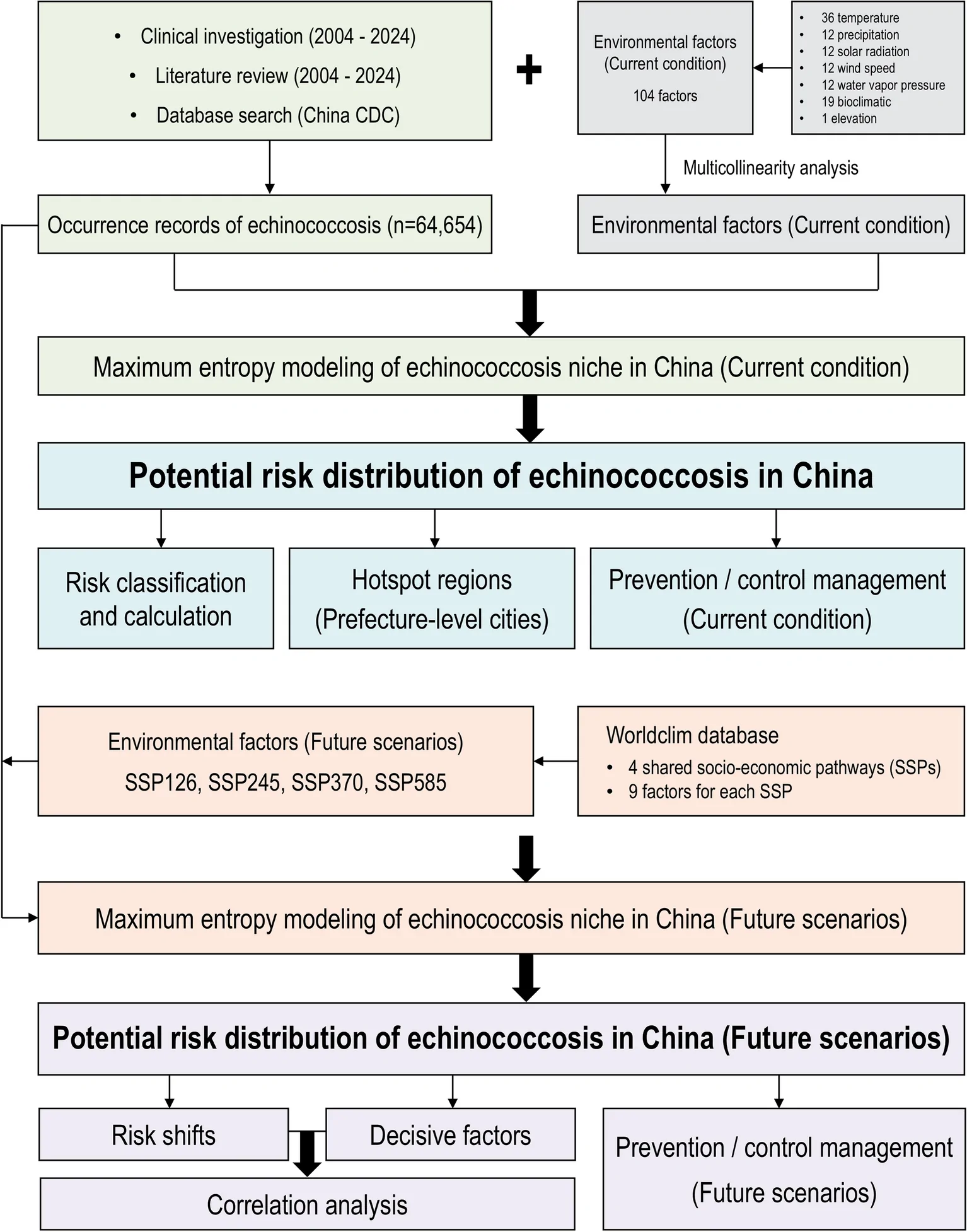
Study framework of the study. The technical route of this study includes five main stages: (1) data collection and processing of echinococcosis incidence; (2) data collection and preparation of environmental factors; (3) Maxent modeling and evaluation; (4) risk calculation and future prediction; and (5) result analysis and visualization

Detailed technical route, including data collection and preprocessing, variable screening, Maxent model construction and evaluation, probability prediction, infection risk calculation, future risk prediction under climate change scenarios, and result analysis and visualization, are displayed in Fig. 3.

## Results

### Model performance and validation

Our model demonstrated good discriminatory power and robustness in predicting echinococcosis transmission risks across China. The model achieved a mean AUC of 0.870 based on 100 bootstrap replicates (Fig. S2), indicating a high level of accuracy in distinguishing between presence and background locations. Further validation based on the independent 25% test dataset yielded a mean CBI close to 1.0 and a high mean TSS value, indicating excellent predictive capacity, stable consistency, and robust transferability of the Maxent model in discriminating echinococcosis suitable habitats (Table S3). Null-model validation, conducted by comparing the performance against models trained on randomly generated pseudo-absence points, confirmed that the predictive capability of our model was statistically significant (*P* < 0.01) and non-random. This robust performance provides a reliable quantitative foundation for all subsequent spatial and temporal risk assessments.

### Key environmental drivers of echinococcosis risk distribution

A comprehensive evaluation of variable contributions, employing both jackknife tests and percent contribution analyses, identified a hierarchy of key environmental factors structuring the potential risk distribution of echinococcosis (Table S2). Elevation (Alt) emerged as the most influential predictor, accounting for 41.4% of the model’s explanatory power. This was followed by annual precipitation (Bio12, 19.7%), precipitation seasonality (Bio15, 10.1%), isothermality (Bio3, 6.4%), and average precipitation in January (Prec1, 5.5%). Further quantitative profiling revealed that regions classified as “very high” risk (risk index ≥ 0.5) are characterized by distinct bioclimatic and topographic signatures (Table 1): a mean elevation of 3292.09 ± 1190.21 m, annual precipitation of 517.59 ± 210.9 mm, high precipitation seasonality (86.8 ± 16.36), moderate isothermality (36.85 ± 5.85), and low winter precipitation (4.61 ± 3.03 mm in January). These parameters collectively define the ecological niche that supports the complex life cycle of *Echinococcus*, influencing host survival, parasite egg persistence, and ultimately, transmission potential.

**Table 1:** Key driving factors of each risk class for echinococcosis distribution under current condition in China

Key driving factor	Low risk	Medium risk	High risk	Very high risk	Unit
Alt	1114.1 ± 1612.07	1861.85 ± 1439.22	2878.55 ± 1597.1	3292.09 ± 1190.21	m
	[− 122 – 6852]	[192 – 6667]	[− 154 – 5116]	[199 – 4935]	
Bio12	789.75 ± 578.24	308.19 ± 323.51	411.35 ± 249.24	517.59 ± 210.9	mm
	[13 – 4256]	[11 – 4434]	[12 – 1846]	[22 – 1403]	
Bio15	89.59 ± 25.18	92.15 ± 20.28	87.19 ± 23.2	86.8 ± 16.36	–
	[20.02 – 152.97]	[28.59 – 145.71]	[24.59 – 141.71]	[22.67 – 143.6]	
Bio3	29.19 ± 6.11	31.97 ± 5.19	33.63 ± 6.62	36.85 ± 5.85	–
	[16.14 – 54.06]	[18.34 – 52.66]	[18.08 – 50.46]	[19.59 – 50.73]	
Prec1	16.99 ± 20.35	4.06 ± 6.83	5.11 ± 4.43	4.61 ± 3.03	mm
	[0 – 286]	[0 – 143]	[0 – 62]	[0 – 21]	
Bio7	38.93 ± 11.26	42.79 ± 7.95	37.84 ± 7.49	35.35 ± 6.58	°C
	[11.52 – 63.72]	[14.78 – 57.54]	[22.34 – 56.66]	[23 – 56.21]	
Prec7	138.96 ± 79.23	70.25 ± 64.86	87.17 ± 51.8	104.21 ± 42.84	mm
	[4 – 962]	[4 – 754]	[3 – 370]	[3 – 276]	
Bio1	8.6 ± 8.89	6.23 ± 6.11	2.64 ± 6.78	2.38 ± 4.56	°C
	[− 19.55 – 25.85]	[− 19.13 – 21.92]	[− 15.68 – 19.62]	[− 14.26 – 16.99]	
Wind6	2.9 ± 0.97	3.05 ± 0.86	3.07 ± 0.8	2.89 ± 0.56	m/s
	[0.9 – 8.01]	[1.08 – 7.09]	[1.1 – 5.49]	[1.11 – 4.95]	
Srad1	8600.14 ± 1994.29	8975.33 ± 1490.82	9304.04 ± 2109.49	9403.3 ± 1651.71	kJ/m^2^/day
	[3541 – 16183]	[5130 – 15319]	[4614 – 14771]	[4339 – 14025]	
Bio14	13.63 ± 15.67	2.93 ± 4.27	4.12 ± 3.23	3.86 ± 2.72	mm
	[0 – 202]	[0 – 95]	[0 – 21]	[0 – 19]	

The values in the table represent mean ± standard deviation. The values in parentheses represent range. “–” represents no unit. Alt—Altitude; Bio1—Annual mean temperature; Bio3—Isothermality; Bio7—Temperature annual range; Bio12—Annual precipitation; Bio14—Precipitation of driest month; Bio15—Precipitation seasonality; Prec1—Average monthly precipitation in January; Prec7—Average monthly precipitation in July; Srad1—Solar radiation of January; Wind6—Wind speed of June

### High-resolution spatial pattern of echinococcosis infection risk

**Fig. 4:**
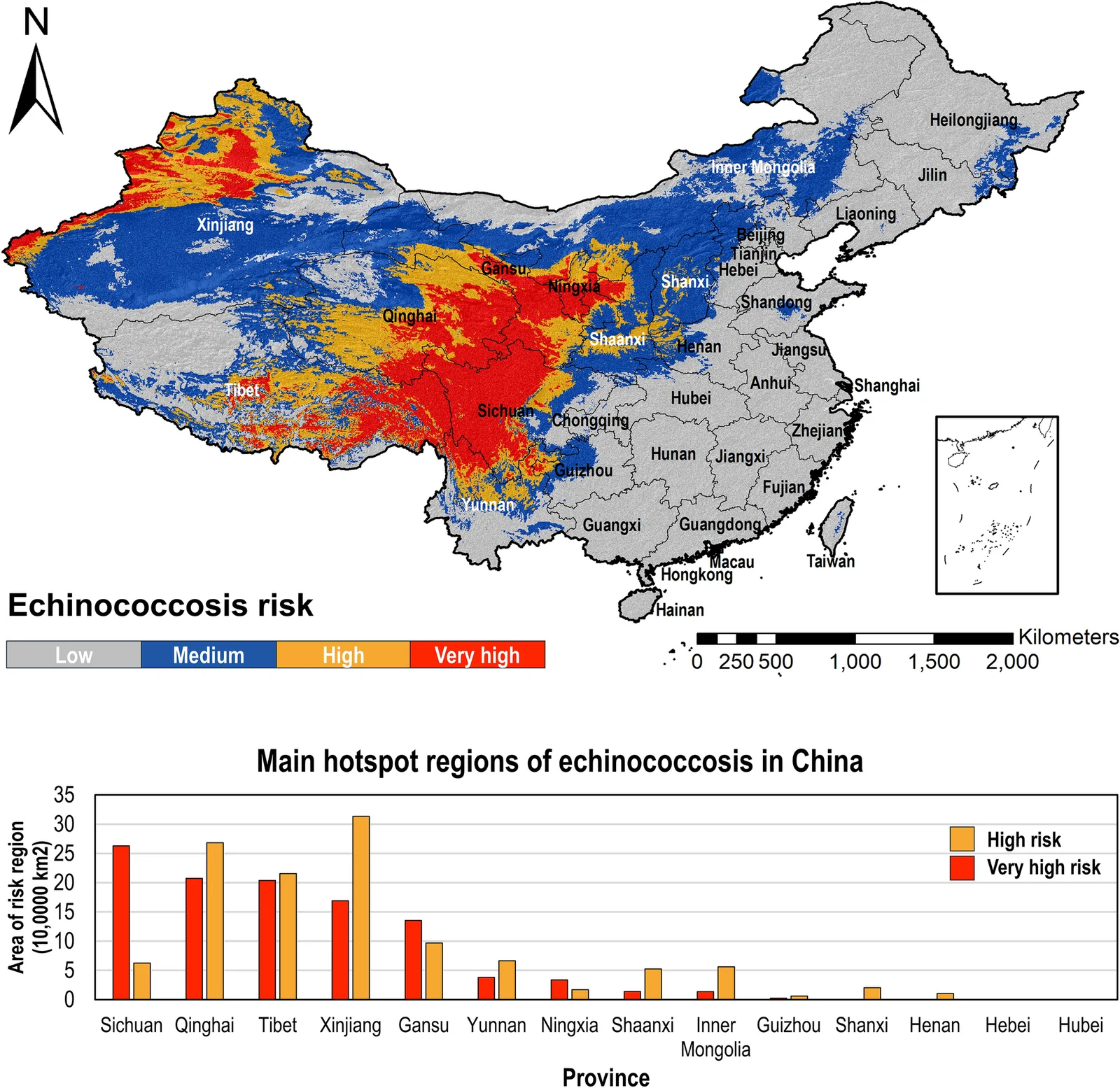
High-resolution distribution map of echinococcosis infection risk under current condition in China. Distribution map showing the potential spatial distribution of echinococcosis infection risk in 34 provincial-level regions of China with different risk levels. Histogram showing area of high- and very high-risk classes in main hotspot regions of echinococcosis in China

**Fig. 5:**
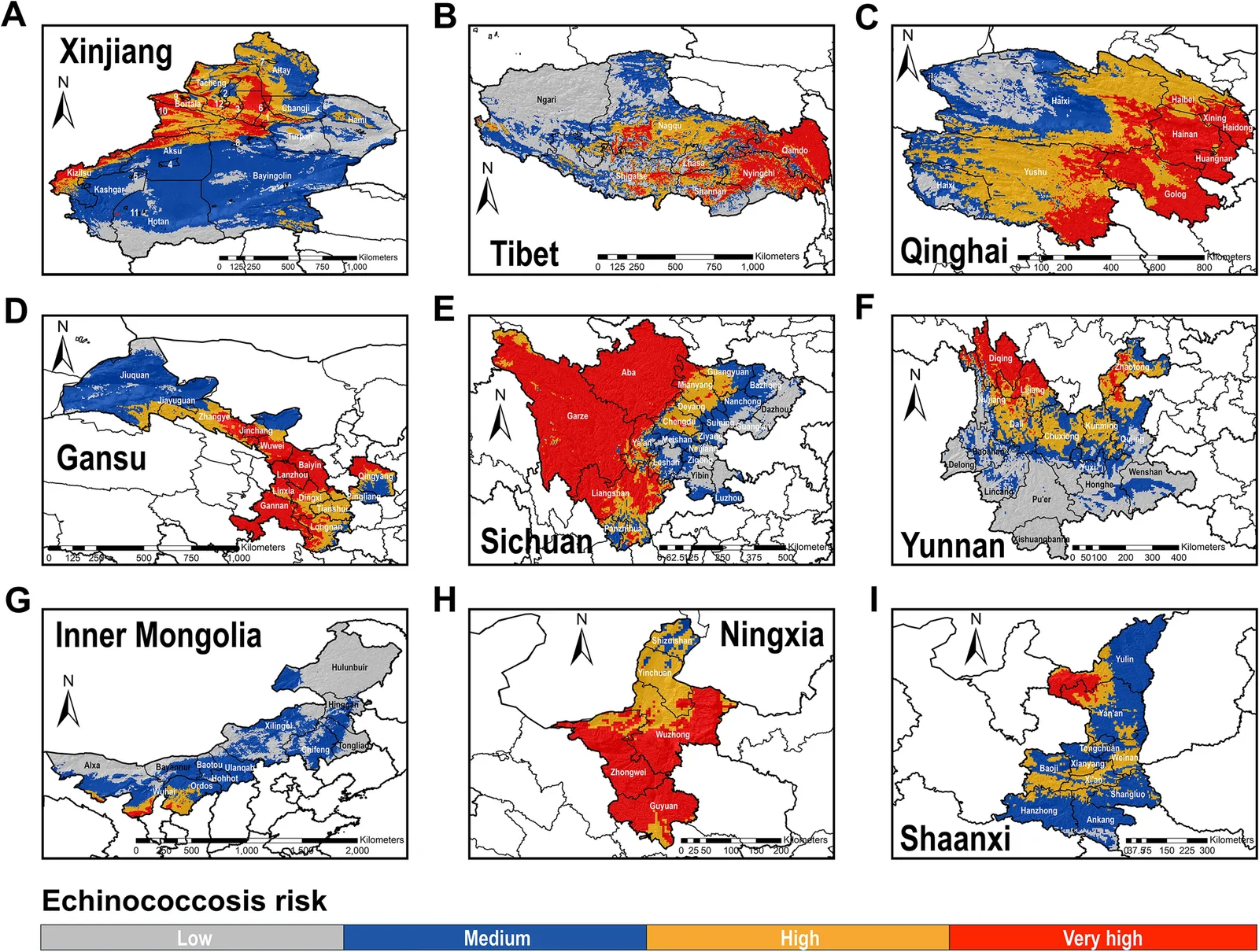
Spatial distribution of echinococcosis risk across prefecture-level cities in major hotspot provinces in China. The spatial distribution pattern of echinococcosis risk in each class (low, medium, high, very high) at the prefecture-level city scale in main hotspot provinces in China are illustrated. **A** Xinjiang Uygur Autonomous Region. **B** Xizang Autonomous Region. **C** Qinghai Province. **D** Gansu Province. **E** Sichuan Province. **F** Yunnan Province. **G** Inner Mongolia Autonomous Region. **H** Ningxia Hui Autonomous Region. **I** Shaanxi Province

Based on the modeling outputs, we generated a high-resolution (30-arcsecond) map of echinococcosis infection risk under current condition (Fig. 4; Table 2). The results delineate a significant and geographically concentrated risk pattern. High and very high-risk regions are overwhelmingly clustered in the western and northern pastoral and agro-pastoral regions of China, spanning the provinces and autonomous regions of Qinghai, Tibet, Xinjiang, Sichuan, Gansu, Yunnan, Ningxia, Inner Mongolia, and Shaanxi. The aggregate area of these hotspot regions covers 226.74 × 10^4^ km^2^, constituting 23.6% of the nation’s total land area. At the provincial level, Qinghai, Tibet, and Xinjiang bear the largest extents of very high-risk area. Drilling down to the prefectural scale reveals critical hotspots (Fig. 5; Table 3): within Tibet, Qamdo Prefecture exhibits the most extensive very high-risk region (81,024 km^2^), followed by Nyingchi (54,253 km^2^); in Qinghai, Golog Tibetan Autonomous Prefecture (58,507 km^2^) and Yushu Tibetan Autonomous Prefecture (47,535 km^2^) are primary foci; and in Xinjiang, Ili Prefecture (28,837 km^2^) and Tacheng Prefecture (27,465 km^2^) represent key risk zones. These regions are universally characterized by a confluence of extensive livestock-based economies, relative socioeconomic underdevelopment, constrained healthcare accessibility, and close human–animal–environment interfaces, factors that synergistically amplify the force of infection.

**Table 2:** Area of different risk classes of echinococcosis in each province of China (10,000 km^2^)

Province	Low risk	Medium risk	High risk	Very high risk
Anhui	13.3576	–	–	–
Beijing	1.3993	0.3403	–	–
Chongqing	7.1910	0.5382	–	–
Fujian	10.7969	–	–	–
Gansu	1.6597	16.5712	9.6753	13.5330
Guangdong	15.4583	–	–	–
Guangxi	20.9236	–	–	–
Guizhou	11.2083	3.9097	0.6111	0.2517
Hainan	2.8698	–	–	–
Hebei	12.9566	6.5000	0.0556	–
Heilongjiang	50.3681	3.8698	–	–
Henan	11.1319	3.9462	1.0712	–
Hong Kong	0.1649	–	–	–
Hubei	16.0243	1.5069	0.0035	–
Hunan	19.4028	–	–	–
Inner Mongolia	66.8438	55.1198	5.6198	1.3819
Jiangsu	9.6806	0.0017	–	–
Jiangxi	15.2760	–	–	–
Jilin	19.7951	1.4983	–	–
Liaoning	14.9965	0.5399	–	–
Macao	0.0033	–	–	–
Ningxia	–	0.2014	1.6910	3.3889
Qinghai	8.6302	15.1927	26.8229	20.7309
Shaanxi	0.4201	13.3333	5.2517	1.3872
Shandong	14.4028	0.8368	–	–
Shanghai	0.4757	–	–	–
Shanxi	0.0590	13.8646	2.0417	-
Sichuan	5.3351	7.6250	6.2465	26.3021
Taiwan	2.9427	0.1615	–	–
Tianjin	1.2222	–	–	–
Tibet	44.3785	27.9670	21.5573	20.3872
Xinjiang	32.3767	94.7604	31.3559	16.9201
Yunnan	15.6042	8.2066	6.6476	3.8021
Zhejiang	9.1979	–	–	–
Total	456.5535	276.4913	118.6511	108.0851

“–” represents an area of 0 under this risk level

**Table 3:** Area of different risk classes of echinococcosis in prefecture-level cities across major hotspot provinces in China (10,000 km^2^)

Hotspot province Prefecture-level city	Low risk	Medium risk	High risk	Very high risk
Xinjiang				
Urumqi	–	0.0278	0.6233	0.8976
Karamay	–	0.5087	0.2170	0.1007
Turpan	4.2135	2.4167	0.7361	0.2674
Hami	9.7969	4.2083	0.9340	0.0052
Aksu	0.3524	10.2431	1.6997	1.4271
Kashgar	2.7500	8.3299	0.1892	0.0087
Hotan	9.1372	15.8628	–	0.0382
Tacheng	–	1.3490	6.5313	2.7465
Altay	0.0017	6.1510	7.1233	0.4965
Changji	0.2604	3.1146	2.6441	2.3316
Bortala	–	0.1111	1.2917	1.3420
Bayingolin	5.5139	38.0243	3.6875	1.7344
Kizilsu	0.0243	3.1007	2.0122	2.1111
Ili	–	0.0399	3.1788	2.8837
Tibet				
Lhasa	0.5590	0.6319	1.0486	0.5278
Shigatse	5.9635	5.3125	3.9045	1.4601
Qamdo	0.0642	0.9219	1.1372	8.1024
Nyingchi	1.0694	1.5313	2.5938	5.4253
Shannan	2.1580	1.5156	2.0434	1.5330
Nagqu	8.1493	12.7969	9.7448	3.2135
Ngari	26.0885	5.2431	1.0660	0.0677
Qinghai				
Xining	–	–	0.1424	0.6302
Haidong	–	–	0.0712	1.2361
Haibei	–	0.0122	2.6632	0.6545
Huangnan	–	–	0.0417	1.7031
Hainan	–	0.0035	0.5573	3.8021
Golog	0.0069	0.0122	1.3542	5.8507
Yushu	0.3125	2.7969	12.0625	4.7535
Haixi	7.7708	10.9670	9.4740	1.9444
Sichuan				
Chengdu	–	0.3559	0.7778	0.2066
Zigong	0.2153	0.1927	–	–
Panzhihua	–	0.3351	0.3125	0.0278
Luzhou	0.5747	0.5174	0.0035	-
Deyang	–	0.1181	0.3993	0.0486
Mianyang	–	0.1927	1.0833	0.6354
Guangyuan	–	0.7691	0.6597	0.1007
Suining	0.1493	0.3472	–	–
Neijiang	0.2934	0.1736	0.0347	–
Leshan	0.3316	0.5694	0.1615	0.1198
Nanchong	0.3420	0.7743	0.0677	0.0035
Meishan	0.1215	0.4809	0.0573	0.0156
Yibin	0.7135	0.4983	0.0087	–
Guangan	0.5260	0.0469	–	–
Dazhou	1.4219	0.1267	–	–
Ya'an	0.0260	0.2951	0.4167	0.6649
Bazhong	0.3924	0.7674	–	–
Ziyang	0.1389	0.3524	0.0434	–
Aba	–	0.0174	0.0139	7.8785
Ganzi	–	0.0451	0.7083	13.1510
Liangshan	0.0104	0.5729	1.4931	3.4132
Gansu				
Lanzhou	–	–	0.0139	1.2882
Jiayuguan	–	0.1267	–	–
Jinchang	–	0.1111	0.1667	0.5017
Baiyin	–	–	0.0399	1.9444
Tianshui	–	0.0694	1.3073	0.0208
Wuwei	0.0156	1.3819	0.5191	1.3767
Zhangye	–	0.4653	2.3906	1.1215
Pingliang	–	0.4010	0.5174	0.1632
Jiuquan	1.6111	12.4878	1.0799	–
Qingyang	–	0.9444	0.7500	0.9653
Dingxi	–	–	0.9184	1.0365
Longnan	–	0.3576	1.4514	0.8958
Linxia	–	–	0.1701	0.6337
Gannan	–	–	0.1024	3.3993
Yunnan				
Kunming	0.0538	0.7882	1.0260	0.0052
Qujing	0.6858	1.0729	0.6667	0.1250
Yuxi	0.8438	0.4774	0.0017	-
Baoshan	1.0000	0.6111	0.0503	-
Zhaotong	0.0260	0.5868	1.0069	0.3906
Lijiang	0.0069	0.1823	0.6910	0.9861
Pu'er	3.8003	0.1024	-	-
Lincang	1.5330	0.5330	0.0052	-
Chuxiong	0.1615	1.1059	1.2795	-
Dali	0.1632	0.8559	1.3559	0.1667
Honghe	2.0642	0.7170	0.0278	-
Wenshan	2.1111	0.6597	-	-
Xishuangbanna	1.6493	-	-	-
Dehong	0.9583	0.0191	-	-
Nujiang	0.4514	0.2899	0.3125	0.2205
Diqing	0.0226	0.0538	0.1944	1.8212
Inner Mongolia				
Hohhot	0.0295	1.7882	-	-
Baotou	0.5556	2.4306	-	-
Hulunbuir	28.0295	3.1076	-	-
Hinggan	3.7622	2.6215	-	-
Tongliao	4.3559	2.1788	-	-
Chifeng	2.9948	6.5938	-	-
Xilingol	10.2569	12.0972	-	-
Ulanqab	0.8472	5.0104	-	-
Ordos	0.0278	5.0451	3.6736	0.2778
Bayannur	3.0573	3.9514	-	-
Wuhai	-	0.1528	0.0122	-
Alxa	12.4826	9.8785	1.9097	1.0903
Ningxia				
Zhongwei	-	-	0.1875	1.1753
Yinchuan	-	0.0365	0.6215	0.0573
Wuzhong	-	-	0.4497	1.2049
Shizuishan	-	0.1632	0.2552	-
Guyuan	-	-	0.1302	0.8941
Shaanxi				
Xi'an	-	0.3229	0.6632	-
Tongchuan	-	0.3142	0.0694	-
Baoji	-	0.8403	0.9097	0.0017
Xianyang	-	0.6354	0.3733	-
Weinan	-	0.5903	0.6840	-
Yan'an	-	2.1823	1.0278	0.4948
Hanzhong	0.0868	2.1875	0.3021	-
Yulin	-	2.8576	0.5903	0.8576
Ankang	0.3281	1.6944	0.2083	-
Shangluo	-	1.5903	0.2865	-

“–” represents an area of 0 under this risk level

### Future spatiotemporal dynamics under climate change scenarios

**Fig. 6:**
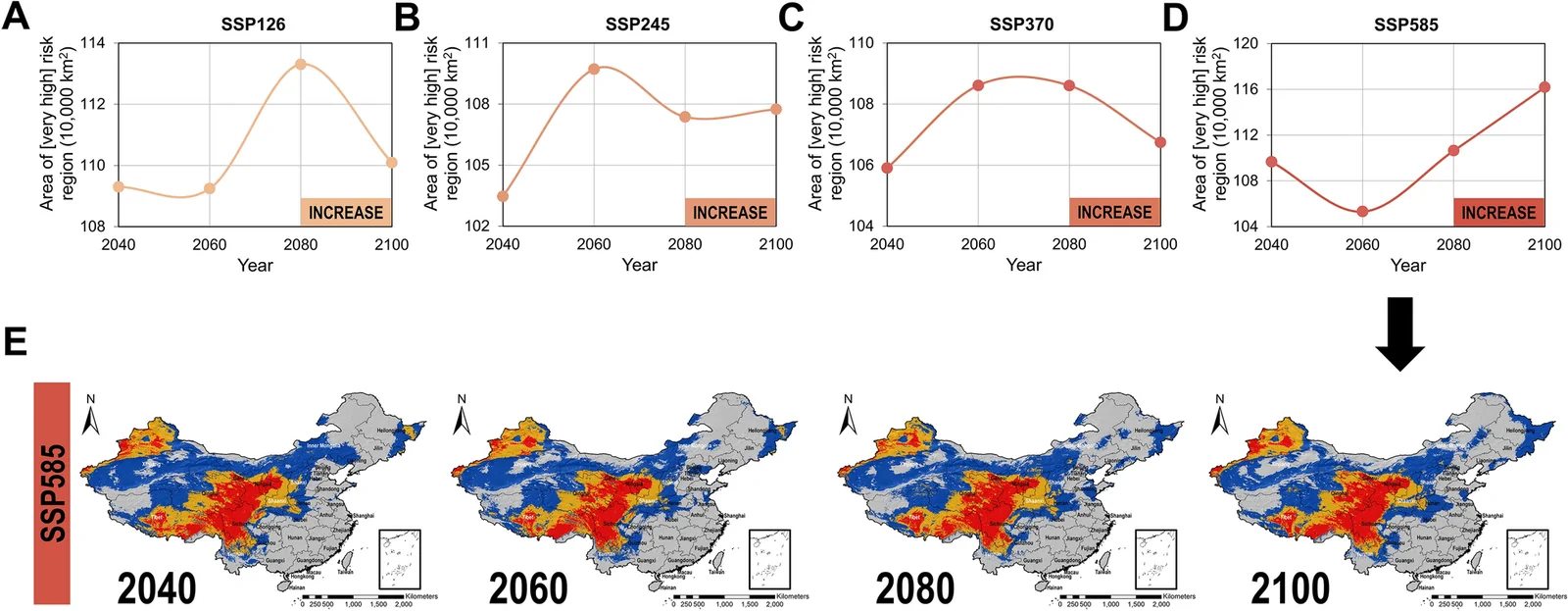
Distribution shifts of echinococcosis infection risk under future climate change scenarios in China. Distribution shifts of very high-risk regions for echinococcosis infection in China from 2040 to 2100 under four representative climate change scenarios. **A** Area shift of very high-risk regions under SSP126 scenario; **B** area shift of very high-risk regions under SSP245 scenario; **C** area shift of very high-risk regions under SSP370 scenario; **D** area shift of very high-risk regions under SSP585 scenario; **E** representative spatial distribution changes of very high-risk regions under SSP585 scenario from 2040 to 2100. SSP, shared socioeconomic pathways

Future projections under four shared socioeconomic pathways (SSP126, SSP245, SSP370, SSP585) from 2040 to 2100 reveal a consistent and concerning trend of geographical expansion for areas conducive to *Echinococcus* transmission (Figs. 6 and S3; Table S4). The magnitude of expansion is strongly correlated with the intensity of the greenhouse gas emission scenario. Under the high-emission SSP585 pathway, the area of “very high” risk is projected to increase by 5.95% by the end of the century (2081–2100), expanding from 1.09 × 10^6^ km^2^ (2021–2040) to 1.16 × 10^6^ km^2^ (2081–2100). This expansion is not merely areal but also directional, indicating a potential eastward and southward shift of suitability zones. In contrast, the low-emission SSP126 scenario forecasts a more moderate 0.72% increase in very high-risk region. The intermediate scenarios, SSP245 and SSP370, project increases of 4.14% and 0.79%, respectively. These projections underscore a direct and positive relationship between future climate forcing and the spatial amplification of echinococcosis transmission risk.

### Climatic mechanisms underpinning future risk shift

**Fig. 7:**
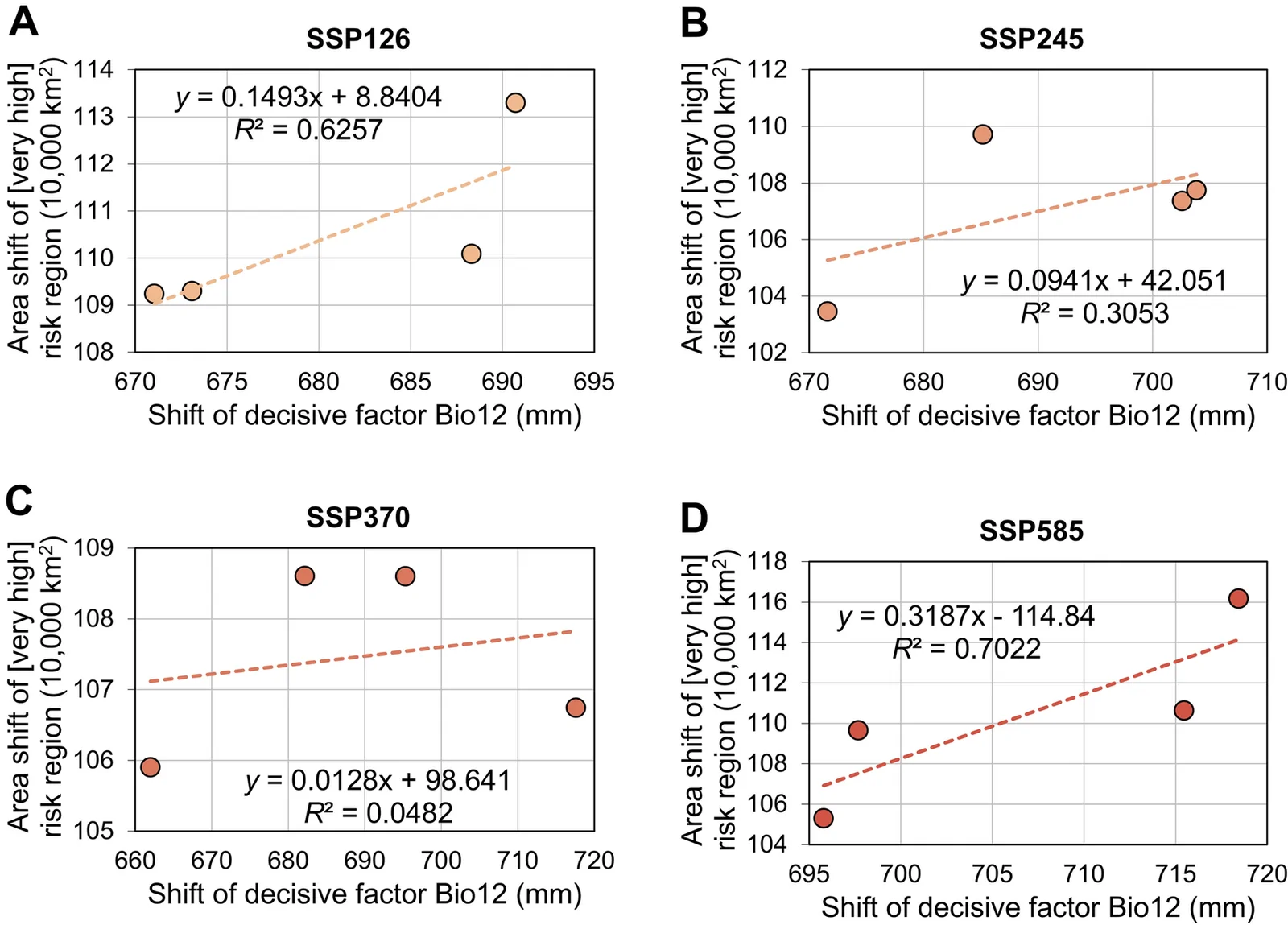
Correlations between changes in echinococcosis risks and changes of key environmental factor Bio12 under different future climate change scenarios. Correlative relationships between the area changes in very high-risk regions of echinococcosis and the changes of Bio12 (annual precipitation, the first key bioclimatic driver identified in this study) across four future climate change scenarios (SSP126, SSP245, SSP370, SSP585). **A** Correlation between area changes of very high-risk regions and Bio12 changes under SSP126 scenario; **B** correlation between area changes of very high-risk regions and Bio12 changes under SSP245 scenario; **C** correlation between area changes of very high-risk regions and Bio12 changes under SSP370 scenario; **D** correlation between area changes of very high-risk regions and Bio12 changes under SSP585 scenario. Each panel presents scatter plots with trend lines, where the *x*-axis represents the magnitude of Bio12 change (mm) and the *y*-axis represents the area changes of very high-risk regions (10,0000 km^2^) from 2040 to 2100. Correlation coefficients (*R*^2^) are indicated in each subplot

To elucidate the mechanisms behind the projected shifts, we analyzed the correlation between the spatiotemporal migration of high-risk region boundaries and changes in key climatic variables. Results identified annual precipitation (Bio12) as the strongest correlate of risk shift across all future scenarios (Fig. 7). The relationship was most pronounced under the SSP126 (*R*^2^ = 0.6257) and SSP585 (*R*^2^ = 0.7022) scenarios. This finding mechanistically links future risk dynamics to alterations in hydrological regimes. In the arid and semi-arid landscapes that dominate China’s high-risk west and north, precipitation is a master regulator of primary productivity, water availability, and host population dynamics. Climate change-induced alterations in precipitation amount, seasonality, and extreme events are, therefore, poised to fundamentally reshape the ecological suitability of these regions for the *Echinococcus* transmission cycle, driving the observed patterns of risk migration.

## Discussion

This study addresses a key limitation of prior provincial‑scale, low‑resolution assessments of echinococcosis in China through the development and validation of a high‑resolution predictive model (AUC = 0.870). The model maps transmission risk at a 30‑arcsecond spatial granularity based on disease incidence data and environmental factors, thereby revealing fine‑scale heterogeneities in risk that were previously unresolved. This analytical framework, which has been successfully validated in our previous studies on main zoonotic diseases in China [20], offers robust ecological interpretability. Rigorous jackknife analysis and percent contribution assessment identified elevation (41.4% contribution) and precipitation patterns (Bio12: 19.7% contribution) not merely as statistical covariates, but as fundamental bioclimatic determinants governing the distribution of *Echinococcus* hosts and the persistence of parasite eggs. Consistent with the results of jackknife tests and percentage contribution analysis, elevation and annual precipitation (Bio12) were confirmed as the most dominant environmental factors shaping the spatial pattern of echinococcosis infection risk. This finding is well-supported by existing landscape epidemiology and One Health-related studies focusing on the Qinghai–Tibet Plateau, arid northwest China and Central Asian endemic regions [6, 28]. Numerous field surveys and spatial simulation studies have verified that elevation gradients profoundly restrict the geographical distribution, habitat selection and population dynamics of definitive and intermediate hosts of *Echinococcus* parasites [29, 30]. Meanwhile, precipitation conditions determine regional microclimatic humidity levels, directly affecting the in vitro survival activity and infectivity of parasite eggs, and further regulating vegetation coverage and food resource availability that modulate the abundance of livestock and wild host populations [31, 32]. In this context, elevation and precipitation are not merely statistical predictive variables, but core ecological drivers that regulate the complete transmission chain of echinococcosis, which reasonably explains their decisive roles in determining disease prevalence and spatial aggregation in alpine pastoral areas. Beyond static risk mapping, the framework establishes a scalable foundation for a dynamic national surveillance system. By integrating real‑time climate anomalies, livestock movement data, and human case reports, such a system could evolve into a precision public health tool, thus enabling a paradigm shift from reactive resource allocation toward evidence‑based, proactive intervention in anticipated transmission hotspots.

A core finding of this study is the potent synergy between environmental suitability and socioeconomic vulnerability in amplifying echinococcosis risk. The high-risk zones identified in western and northern China (Qinghai, Tibet, Xinjiang, Inner Mongolia, etc.) are characterized not only by ecologically conducive conditions (e.g., mean elevation of 3292.09 m, annual precipitation of 517.59 mm for very high-risk areas) but also by profound socioeconomic marginalization: low per capita GDP, inadequate healthcare infrastructure, and heavy reliance on livestock husbandry that necessitates frequent human–animal contact [28, 33–35]. This confluence creates a self-reinforcing “poverty-disease trap”: environmental conditions favor pathogen transmission, while structural barriers limit access to timely diagnosis, treatment, and preventive veterinary services [36, 37]. For high-risk occupational groups (herders, veterinarians), this nexus of ecological suitability and occupational exposure exacerbates infection risk, which is quantified in our prefecture-level mapping as concentrated very high-risk areas in pastoral regions. Critically, this finding demonstrates that echinococcosis, like many neglected zoonoses, is a product of intertwined ecological and social determinants. Effective interventions must, therefore, adopt a dual-focus approach: targeting biological transmission pathways (e.g., host management, environmental decontamination) while addressing the underlying social determinants of health inequity (e.g., improving healthcare access, diversifying livelihoods in pastoral communities).

The stark spatial concentration of echinococcosis risk, encompassing 23.6% of China’s landmass yet disproportionately burdening marginalized pastoral populations, graphically positions the disease as a “poverty-related zoonosis”. Beyond the catastrophic clinical toll (e.g., 94% 5-year mortality for advanced alveolar echinococcosis), the disease inflicts a crippling economic burden (over 1.3 billion RMB annually) through medical expenditures and livestock losses, further entrenching poverty in affected communities. Our prefecture-level risk mapping reveals profound intra-provincial disparities (e.g., Qamdo Prefecture in Tibet boasts the largest very high-risk area at 81,024 km^2^; Golog Tibetan Autonomous Prefecture in Qinghai follows at 58,507 km^2^), directly challenging the efficacy of uniform, one-size-fits-all control policies. Notably, the spatial overlap between high-burden areas and national rural revitalization priorities presents a strategic opportunity [6, 36]. Embedding echinococcosis control within broader initiatives to improve rural infrastructure, health education, veterinary services, and universal healthcare access can disrupt the poverty–disease cycle, aligning disease elimination with sustainable development goals and enhancing health equity.

Our future projections deliver a clear and urgent warning: climate change will significantly amplify echinococcosis transmission risk, with the magnitude of impact tightly coupled to global greenhouse gas emission trajectories. Under the high-emission SSP585 scenario, the area of very high-suitability for echinococcosis transmission is projected to increase by 5.95% by 2081–2100 (from 1.09 × 10^6^ km^2^ to 1.16 × 10^6^ km^2^), with a significant eastward and southward expansion of risk zones. In contrast, the low-emission SSP126 scenario limits this expansion to 0.72%, highlighting the critical role of climate mitigation in reducing future disease burden. Mechanistically, our analysis identifies annual precipitation (Bio12) as the strongest correlate of risk redistribution (*R*^2^ = 0.7022 under SSP585), reflecting its role as a master regulator of primary productivity, water availability, and host population dynamics in arid and semi-arid high-risk regions. These findings call for a shift in control planning toward adaptive, climate-informed frameworks and away from static, climate-agnostic strategies. Future interventions should incorporate climate resilience measures, such as precipitation-triggered early-warning systems, adaptive livestock grazing practices, and targeted surveillance in newly emerging risk zones.

While this study provides actionable insights through the integration of high-resolution ecological modeling and CMIP6 climate projections, several limitations warrant acknowledgment and future refinement. First, potential under-reporting of echinococcosis cases in remote pastoral areas may introduce sampling bias, despite efforts to mitigate this through spatial filtering and multi-source data integration (clinical surveys, peer-reviewed literature, CDC records). Second, the correlative nature of the Maxent model, while robust for distributional modeling, cannot establish causal relationships; complementary experimental studies (e.g., laboratory assessments of parasite egg survival under varying temperature/precipitation regimes) are needed to validate the ecological mechanisms identified here. Third, future projections rely on a single global climate model (BCC–CSM2–MR); multi-model ensemble approaches would better quantify uncertainty associated with climate scenario simulations. Fourth, the use of static socioeconomic variables (e.g., baseline GDP, healthcare access) limits the ability to project how changing socioeconomic conditions (e.g., rural development, urbanization) may modulate future risk. Addressing these limitations will require interdisciplinary collaboration: field validation of environmental drivers, integration of dynamic socioeconomic datasets, and adoption of multi-model climate projections to enhance the robustness of future risk assessments.

Translating these findings into tangible public health gains requires their integration into a cohesive One Health policy framework, one that bridges human health, animal health, environmental management, and meteorological sectors. Key priorities include: (1) institutionalizing climate-informed surveillance systems to monitor emerging risk zones; (2) implementing geographically and socially targeted interventions in high-burden communities (e.g., mass dog deworming, health education for herders, improved access to diagnostic tools); (3) promoting climate-resilient livestock practices to reduce human–animal–pathogen contact; and (4) strengthening cross-sectoral collaboration and regional cooperation to address transboundary echinococcosis risks with neighboring endemic countries.

In addition, from the perspective of technology translation and practical application, the current modeling framework can be evaluated using the standard technology readiness level (TRL) classification. The main findings of this study are currently at TRL 4, representing technology validation under laboratory and computational analytical environments. We have completed model construction, internal performance validation, spatial pattern verification, and future climate scenario simulation based on multi-source epidemiological and environmental datasets, which further confirms the robust predictive performance and ecological interpretability of the model. At present, this study has realized theoretical algorithm modeling and offline spatiotemporal simulation analysis. In the future, we plan to carry out field verification in typical endemic pastoral areas, integrate real-time climate monitoring, livestock distribution, and human case surveillance data, and further upgrade the model to TRL 5–6 to support practical demonstration and precise intervention for regional echinococcosis prevention and control.

In summary, this study provides a rigorous, spatially explicit, and temporally dynamic assessment of echinococcosis risk in China. It clarifies the current risk landscape shaped by poverty–environment synergies in western and northern regions, identifies key climatic drivers, and projects a concerning expansion of risk under climate change (dependent on global emission choices). By adopting a One Health lens that integrates ecological niche modeling and climate futures, this work offers not just a diagnosis of the problem but a science-based blueprint for action. Achieving echinococcosis elimination targets and mitigating future risk will require integrated, adaptive, and equity-focused strategies, ones that are as complex and interconnected as the disease transmission system itself. This framework serves as a transferable model for confronting the growing challenge of neglected zoonoses in an era of global environmental and socioeconomic change.

## Conclusions

This study provides a rigorous, spatially explicit assessment of echinococcosis risk in China via a One Health framework and Maxent model. Key findings reveal high/very high-risk hotspot regions (23.6% of national land) concentrated in western/northern pastoral regions (Qinghai, Tibet, Xinjiang, etc.), shaped by elevation, precipitation, and a “poverty-disease trap” of socioeconomic underdevelopment and limited healthcare. Future projections under four climate scenarios show concerning risk expansion (5.95% increase in very high-risk areas by 2100 under SSP585) driven primarily by precipitation shifts. Low-emission pathways (SSP126) mitigate this expansion. These results offer a science-based blueprint for targeted, adaptive interventions, integrating disease control with rural revitalization and climate resilience, while providing a transferable model for neglected zoonoses in climate-vulnerable regions.

## Supplementary Information

Supplementary Material 1.

## Data Availability

All data relevant to the study are included in the article or uploaded as online supplementary information.

## Abbreviations

CMIP6: Coupled model intercomparison project phase 6
AUC: Area under the receiver operating characteristic curve
Maxent: Maximum entropy
SSP: Shared socioeconomic pathway
