# Supplementary material for: Risk prediction of echinococcosis in China under climate change: a One Health approach
Source: Parasit Vectors. 2026 Jun 18;19:366. doi: 10.1186/s13071-026-07518-7 (PMC13520399; doi:10.1186/s13071-026-07518-7)
Supplement: Supplementary file 1 [file 13071_2026_7518_MOESM1_ESM.docx]

**Supplementary Information for**

Risk prediction of echinococcosis in China under climate change: A One Health approach

Tengyue Diao^1,2^, Nan Xu^3^, Ning Ma^2^, Haiyan Chen^2^, Na Wang^2^, Ting La^4^, Kunyi Wu^2^, Bo Cao^1,2,5^

^1^ Department of Infectious Diseases, The Second Affiliated Hospital of Xi'an Jiaotong University, Xi'an 710004, China

^2^ Core Research Laboratory, The Second Affiliated Hospital of Xi'an Jiaotong University, Xi'an 710004, China

^3^ Department of Clinical Laboratory, The Second Affiliated Hospital of Xi'an Jiaotong University, Xi'an 710004, China

^4^ National-Local Joint Engineering Research Center of Biodiagnosis & Biotherapy, The Second Affiliated Hospital of Xi'an Jiaotong University, Xi'an 710004, China

^5^ Key Laboratory of Surgical Critical Care and Life Support (Xi'an Jiaotong University), Ministry of Education, Xi'an 710004, China

Running title: Echinococcosis risk in China

*Address for correspondence:

Bo Cao, Ph.D., Professor

The Second Affiliated Hospital of Xi'an Jiaotong University

157 West Fifth Road

Xi’an 710004, China

Email: [bo_cao@xjtu.edu.cn](mailto:bo_cao@xjtu.edu.cn)

ORCID: <https://orcid.org/0000-0003-4656-7152>

**This file includes:**

*Supplementary Figure 1-3*

*Supplementary Table 1-4*

**Figure S1. Workflow of echinococcosis occurrence data collection via systematic literature review.**

This figure illustrates the systematic workflow employed to collect echinococcosis occurrence data through comprehensive literature review, ensuring the robustness and representativeness of the epidemiological dataset used in this study. The review and screening process consisted of five consecutive stages: (1) Review strategy formulation: Core keywords were determined based on the research focus, including English terms (“echinococcosis”, “hydatid disease”, “*Echinococcus granulosus*”, “*Echinococcus multilocularis*”, “China”, “case report”, “epidemiological survey”) and corresponding Chinese terms. A combined retrieval formula was constructed using logical operators (AND/OR) to optimize retrieval comprehensiveness. (2) Database selection and initial retrieval: Literature was retrieved from both international and Chinese academic databases, including PubMed, Web of Science, China National Knowledge Infrastructure (CNKI), Wanfang Data Knowledge Service Platform, and VIP Chinese Journal Database. The retrieval period was set from January 2004 to December 2024 to ensure no recent studies were omitted. (3) Duplicate removal: Retrieved literature records were imported into EndNote X9 software, and duplicate studies were automatically identified and removed based on title, author, and publication year; manual checking was further performed to eliminate missed duplicates. (4) Eligibility screening: Two independent researchers screened the literature in two rounds. First, titles and abstracts were reviewed to exclude irrelevant studies (e.g., non-echinococcosis topics, studies outside China, review articles without original case data). Second, full texts of potentially eligible studies were assessed against predefined inclusion criteria: (a) Original studies reporting echinococcosis case data (incidence, prevalence, or individual case information) in China; (b) Studies with clear geographical information (province, prefecture, or county level); (c) Studies with complete data sources and explicit research methods. Exclusion criteria included: (a) Case reports with incomplete geographical or clinical information; (b) Duplicate publications of the same dataset; (c) Non-peer-reviewed literature (e.g., conference abstracts, dissertations without formal publication); (d) Studies with ambiguous diagnosis criteria for echinococcosis. Discrepancies between the two researchers during screening were resolved through discussion with a third senior researcher. (5) Data extraction and validation: For eligible studies, a standardized data extraction form was used to collect key information, including study basic information (author, publication year, study area), case characteristics (number of cases, type of echinococcosis, age/sex of patients), and epidemiological indicators (incidence rate, prevalence rate). Extracted data were cross-validated by two researchers; inconsistent data were verified by rechecking the original literature, and unresolved issues were consulted with the corresponding authors of the original studies when necessary. The flow diagram also presents the number of literature records obtained at each stage (e.g., initial retrieval results, number after duplicate removal, number after title/abstract screening, number after full-text screening, and final included studies), quantifying the rigor of the data collection process. This systematic literature retrieval workflow ensured that the collected echinococcosis case data were comprehensive, accurate, and geographically explicit, laying a solid foundation for subsequent high-resolution risk mapping and spatiotemporal projection analyses.

**Figure S2. Workflow of echinococcosis occurrence data collection via systematic literature review.**

Jackknife training gain, ROC curve, AUC value and null-model validation for Maxent modeling of echinococcosis risk under current condition. (A): Jackknife results for echinococcosis risk. The Jackknife training gain for echinococcosis risk was used as reference for generating percent contrition of environmental factors in Maxent modeling. (B): ROC curve and AUC value for echinococcosis risk. (C): Null-model validation of Maxent modeling for echinococcosis risk. ROC: receiver operating characteristic. AUC: area under the curve.

**Figure S3. Spatial distribution changes of different echinococcosis risk grades in China under future climate change scenarios.**

Spatiotemporal dynamics of echinococcosis risk across different risk classes (low, medium, high, and very high) in China under four representative future climate change scenarios: SSP126 (low-emission scenario), SSP245 (intermediate-low emission scenario), SSP370 (intermediate-high emission scenario), and SSP585 (high-emission scenario). SSP: shared socioeconomic pathway.

**Table S1.** Moran’s I spatial autocorrelation results for datasets thinned under different distance gradient thresholds

| **Spatial thinning threshold** | **Moran’s I index** | **Z-score** | ***P*-value** | **Spatial autocorrelation** |
| --- | --- | --- | --- | --- |
| 2 km minimum / 10 km maximum | 0.012 | 1.31 | 0.19 | Not significant |
| 2 km minimum / 25 km maximum | 0.008 | 1.12 | 0.263 | Not significant |
| 2 km minimum / 30 km maximum | 0.005 | 0.76 | 0.447 | Not significant |

*Note*: Spatial thinning was performed with a fixed minimum distance of 2 km and three gradient maximum distance thresholds (10 km, 25 km, 30 km). Moran’s I statistic was used to evaluate spatial autocorrelation of echinococcosis occurrence points. A *P*-value > 0.05 indicates no statistically significant spatial autocorrelation, ensuring spatial independence of the filtered dataset. The threshold combination of 2 km minimum and 25 km maximum was finally selected to balance spatial independence and retention of valid endemic occurrence records in sparsely distributed high-altitude pastoral regions of western China.

**Table S2.** Percent contribution and permutation importance of key drivers for risk distribution of echinococcosis under current condition in China

| **Variable** | **Percent contribution** | **Permutation importance** | **Description** |
| --- | --- | --- | --- |
| Alt | 41.4 | 30.5 | Elevation |
| Bio12 | 19.7 | 27.3 | Annual precipitation |
| Bio15 | 10.1 | 1.2 | Precipitation seasonality |
| Bio3 | 6.4 | 4.6 | Isothermality |
| Prec1 | 5.5 | 4.8 | Average monthly precipitation in January |
| Bio7 | 4.7 | 5.3 | Temperature annual range |
| Prec7 | 3.2 | 11.6 | Average monthly precipitation in July |
| Bio1 | 3 | 7.7 | Annual mean temperature |
| Wind6 | 2.2 | 1.1 | Average monthly wind speed in June |
| Srad1 | 2 | 4.4 | Average monthly solar radiation in January |
| Bio14 | 1.7 | 1.5 | Precipitation of the driest month |

**Table S3.** Summary of model validation metrics across 100 bootstrap replicates based on the 25% independent test dataset

| **Validation metric** | **Mean ±**  **Standard deviation** | **Range**  **(Min-Max)** | **Interpretation** |
| --- | --- | --- | --- |
| AUC | 0.870 ± 0.026 | 0.821-0.915 | Excellent discriminatory performance |
| Continuous boyce index (CBI) | 0.924 ± 0.031 | 0.868-0.976 | High predictive consistency and reliability |
| True skill statistic (TSS) | 0.785 ± 0.042 | 0.712-0.853 | Good overall predictive accuracy |

*Note:* All metrics were calculated using the 25% independent test dataset reserved in each of the 100 Maxent bootstrap replicates. AUC represents the area under the receiver operating characteristic curve for the testing partition. The Continuous boyce index (CBI) ranges from -1 to 1; values close to 1 indicate robust model ability to distinguish suitable from unsuitable habitats. True skill statistic (TSS) ranges from 0 to 1, with higher values indicating better overall model discriminatory performance. Mean and standard deviation were summarized across all 100 replicates.

**Table S4.** Risk shift of hotspot regions of echinococcosis distribution under future scenarios in China

| **Shared socioeconomic pathway (SSP)** | **2040** | **2060** | **2080** | **2100** |
| --- | --- | --- | --- | --- |
| SSP126 | 109.3021 | 109.2448 | 113.3021 | 110.0938 |
| SSP245 | 103.4635 | 109.7153 | 107.3681 | 107.7465 |
| SSP370 | 105.9045 | 108.6094 | 108.6024 | 106.7448 |
| SSP585 | 109.6580 | 105.3056 | 110.6354 | 116.1788 |

*Note*: Area of very high risk class of echinococcosis distribution under future scenarios was calculated and shown in table. Unit: 10,000 km^2^.
